# Evaluation of ABCG2-mediated extra-renal urate excretion in hemodialysis patients

**DOI:** 10.1038/s41598-022-26519-x

**Published:** 2023-01-13

**Authors:** Yuki Ohashi, Masao Toyoda, Nobumichi Saito, Masahiro Koizumi, Genta Kanai, Hirotaka Komaba, Moritsugu Kimura, Takehiko Wada, Hiroo Takahashi, Yuichiro Takahashi, Naoto Ishida, Takatoshi Kakuta, Masafumi Fukagawa, Kimiyoshi Ichida

**Affiliations:** 1 Department of Pathophysiology, Tokyo University of Pharmacy and Life Sciences, 1432-1 Horinouchi, Hachioji-Shi, Tokyo, 192-0392 Japan; 2 Division of Nephrology, Endocrinology and Metabolism, Department of Internal Medicine, Tokai University School of Medicine, 143 Shimokasuya, Isehara-Shi, Kanagawa 259-1193 Japan; 3 Division of Nephrology, Endocrinology and Metabolism, Tokai University Oiso Hospital, 21-1 Gakkyo, Oiso-Machi, Naka-Gun, Kanagawa 259-0198 Japan; 4 Jinken Clinic, 15-1 Ogi-Cho, Ebina-Shi, Kanagawa 243-0436 Japan; 5 Seichi Clinic, 5-19-8 Numame, Isehara-Shi, Kanagawa 259-1126 Japan; 6 Division of Nephrology, Endocrinology and Metabolism, Tokai University Hachioji Hospital, 1838 Ishikawa-Machi, Hachioji-Shi, Tokyo, 192-0032 Japan; 7 Division of Kidney and Hypertension, Department of Internal Medicine, Jikei University School of Medicine, 3-19-18 Shinbashi, Minato-Ku, Tokyo, 105-8471 Japan

**Keywords:** Medical research, Nephrology

## Abstract

Two-thirds of urate is excreted via the renal pathway and the remaining one-third via the extra-renal pathway, the latter mainly via the intestine in healthy individuals. ABCG2, a urate exporter, is expressed in various tissues including the kidney and intestine, and its dysfunction leads to hyperuricemia and gout. ABCG2 is regarded as being responsible for most of the extra-renal urate excretion. However, the extra-renal urate excretion capacity via ABCG2 remains undefined in end-stage kidney diseases. Therefore, we evaluated the capacity of extra-renal ABCG2 using 123 anuric hemodialysis patients whose urate excretion depended on only the extra-renal pathway. ABCG2 function in each participant was estimated based on *ABCG2* dysfunctional variants. We computed the uric acid pool (Pool_UA_) from bodyweight and serum urate level (SUA) using previously reported radio-isotopic data, and we analyzed the association between ABCG2 function and the Pool_UA_. SUA and Pool_UA_ increased significantly with ABCG2 dysfunction, and extra-renal ABCG2 could excrete up to approximately 60% of the daily uric acid turnover in hemodialysis patients. Our findings indicate that the extra-renal urate excretion capacity can expand with renal function decline and highlight that the extra-renal pathway is particularly important in the uric acid homeostasis for patients with renal dysfunction.

## Introduction

Serum urate levels (SUAs) are regulated by the balance between production and excretion of uric acid. Urate is excreted via renal and extra-renal pathways, the latter mainly involving the intestinal tract. According to radio-isotope experiments conducted more than half a century ago^1–12^, roughly two-thirds of urate excretion occurs via the renal pathway, and the remaining one-third mainly via the intestine in normal individuals.

In the kidney, uric acid is reabsorbed and secreted after being freely filtered through the glomerulus. Renal urate excretion is regulated by various reabsorptive and secretory transporters in the renal proximal tubules such as SLC22A12, SLC2A9, and ATP-binding cassette subfamily G member 2 (ABCG2)^13–18^. ABCG2, expressed in a variety of tissues including kidneys and intestine, secretes diverse compounds such as urate. In the intestine, uric acid is also excreted by several urate transporters including ABCG2^19–22^. The expression of ABCG2 in the intestine is remarkable compared to other intestinal urate transporters (e.g. SLC2A9, SLC17A4, and ABCC4) (browsed THE HUMAN PROTEIN ATLAS, https://www.proteinatlas.org/, 2022/8/17). Additionally, it has been reported that single nucleotide polymorphisms (SNPs) of *ABCG2* have an order of magnitude greater impact on the SUA and gout than do the SNPs of other urate transporters expressed in the intestine in the general population^23,24^. The impact of *ABCG2* SNPs was even greater in chronic kidney disease patients who have lower renal urate excretion than in the general population^24,25^. Taken together, the above considerations lead us to believe that ABCG2 serves as the predominant transporter in the intestine. Indeed, there are many papers suggesting that ABCG2 is the major exporter in extra-renal urate excretion^26–32^. This study evaluated extra-renal urate excretion by ABCG2 in anuric patients with end-stage renal disease (hemodialysis patients) whose urate excretion depended on only the extra-renal pathway.

Evaluation of uric acid pool size (Pool_UA_) and uric acid production was a secondary aim of this study. Pool_UA_ and uric acid production had previously been measured in radio-isotopic experiments conducted more than half a century ago^1–12^. However, both Pool_UA_ and the amount of uric acid production have not been evaluated since then because of ethical constraints regarding the use of radio-isotopes in human subjects. Recently, animal studies using ^11^C-labeled uric acid as a positron emission tomography (PET) probe have been reported to minimize radiation exposure, but optimization for humans is still in its infancy^33^. Therefore, we considered constructing a design that is as noninvasive as possible to the subjects. Combining data from hemodialysis patients with results from previous radio-isotopic studies, we estimated Pool_UA_ and uric acid production in individuals receiving dietary guidance for hemodialysis.

## Results

### Summary of clinical characteristics in hemodialysis participants

**Figure 1:**
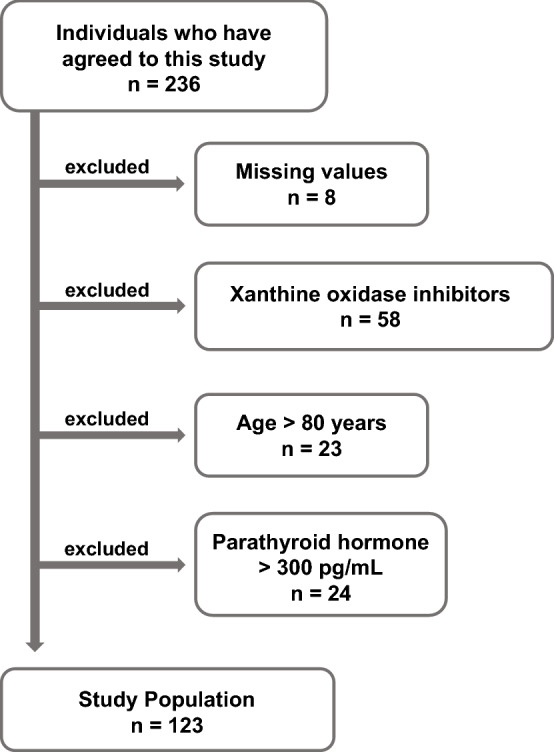
Participant screening flowchart.

In this study, 123 hemodialysis participants were included. A flowchart showing the exclusion criteria for selecting participants for inclusion in this study is shown in Fig. 1. We summarized the clinical characteristics of 123 hemodialysis participants before the hemodialysis with a 2-day interval (Table 1). Overall, the age of the participants was 67.2 ± 10.0 years (mean ± standard deviation), and 74.8% were male. The mean SUA was 7.4 ± 1.1 mg/dL at enrollment of this study (before the hemodialysis with a 2-day interval), and there was no significant difference by sex (male SUA: 7.4 ± 1.1 mg/dL, female SUA: 7.3 ± 1.1 mg/dL). The minor allele frequencies of SNPs, p.Q126X and p.Q141K, of ABCG2 were 1.6% and 19.1%, respectively. Based on the combination of p.Q126X and p.Q141K, the numbers of participants by estimated ABCG2 function were 76 (with 100% ABCG2 function), 39 (with 75% ABCG2 function), and 8 (with 50% ABCG2 function).

**Table 1:** Clinical characteristics of hemodialysis participants.

		Estimated ABCG2 function					P value
	Total	100%		75%		50%	
	n = 123	n = 76		n = 39		n = 8	
Age (years)	67.2 (10.0)	67.4 (10.5)		67.3 (9.3)		64.3 (9.5)	0.698
Male (%)	92 (74.8%)	55 (72.4%)		33 (84.6%)		4 (50.0%)	0.089
Body mass index (kg/m^2^)	22.1 (3.1)	22.1 (3.1)		22.2 (3.1)		22.3 (3.5)	0.979
Systolic blood pressure (mmHg)	152.1 (21.5)	149.8 (22.7)		156.4 (19.2)		152.9 (19.9)	0.313
Diastolic blood pressure (mmHg)	76.3 (13.8)	75.4 (12.8)		78.5 (16.0)		74.6 (13.3)	0.487
Red blood cell (10^6^/μL)	3.6 (0.4)	3.7 (0.4)		3.5 (0.4)		3.6 (0.4)	0.319
Hematocrit (%)	34.4 (3.8)	34.8 (4.1)		33.8 (3.4)		34.2 (3.9)	0.386
Hemoglobin (g/dL)	11.0 (1.1)	11.1 (1.2)		10.8 (1.0)		10.8 (1.1)	0.420
Platelet (10^3^/μL)	196.8 (58.6)	19.7 (5.8)		19.1 (5.9)		22.8 (6.2)	0.261
Serum albumin (g/dL)	3.6 (0.3)	3.6 (0.4)		3.7 (0.2)		3.6 (0.4)	0.915
Blood urea nitrogen (mg/dL)	62.3 (14.3)	63.2 (13.4)		61.4 (15.2)		57.0 (18.6)	0.464
Serum creatinine (mg/dL)	11.0 (2.4)	11.0 (2.4)		11.2 (2.2)		10.3 (2.9)	0.558
Serum urate (mg/dL)	7.4 (1.1)	7.1 (1.0)		7.8 (1.1)		8.4 (1.4)	** < 0.001**
Aspartate aminotransferase (IU/L)	13.1 (6.5)	12.9 (5.5)		13.2 (8.4)		14.9 (4.9)	0.721
Alanine aminotransferase (IU/L)	10.5 (6.1)	10.4 (4.8)		10.8 (8.5)		10.3 (3.8)	0.944
γ-glutamyl transpeptidase (IU/L)	23.6 (25.6)	25.1 (28.5)		20.3 (17.2)		25.0 (31.9)	0.628
Parathyroid hormone (pg/mL)	120 (86)	127.3 (87.5)		114.0 (84.0)		89.6 (73.4)	0.422
Sodium (mEq/L)	139 (3)	139.1 (2.7)		139.5 (3.1)		139.6 (2.1)	0.719
Potassium (mEq/L)	4.8 (0.7)	4.8 (0.7)		4.7 (0.8)		4.8 (0.9)	0.669
Chloride (mEq/L)	102 (4)	101.4 (3.6)		102.5 (4.0)		101.3 (3.8)	0.305
Calcium (mg/dL)	8.6 (0.7)	8.6 (0.8)		8.6 (0.7)		8.9 (0.5)	0.579
Phosphate (mg/dL)	4.9 (1.2)	4.9 (1.1)		4.9 (1.4)		4.3 (1.4)	0.395

Significant values are in bold.

The values represent the mean (standard deviation or proportion) immediately before the start of hemodialysis with a 2-day interval.

### Series of changes in SUA related to hemodialysis

The SUA increases over 2-day and 3-day hemodialysis interval were adjusted by hematocrit (SUA_Ht_) and are shown in Table 2. Over a 2-day hemodialysis interval, the SUA_Ht_ increase with 100%, 75%, and 50% ABCG2 function was 4.56 ± 0.85, 4.65 ± 0.80, and 5.12 ± 1.34 mg/dL (P = 0.223), respectively. Over 3-day hemodialysis interval, the SUA_Ht_ increase with 100, 75 and 50% functional ABCG2 was 5.77 ± 1.01, 6.35 ± 1.00 and 7.10 ± 1.71 mg/dL (P < 0.001), respectively. The SUA_Ht_ increase per 24 h with 100%, 75%, and 50% ABCG2 function, calculated by the difference between the SUA_Ht_ increase over 2 days and 3 days, was 1.20 ± 0.69, 1.71 ± 0.74 and 1.97 ± 0.67 mg/dL/day (P < 0.001), respectively.

**Table 2:** Changes in serum urate levels related to hemodialysis.

	ABCG2 functional category			P value
	100%	75%	50%	
**Hemodialysis with a 2-day interval**				
Pre-hemodialysis SUA (mg/dL)	7.1 (1.0)	7.8 (1.1)	8.4 (1.4)	** < 0.001**
Post-hemodialysis SUA (mg/dL)	2.0 (0.5)	2.2 (0.6)	2.4 (0.7)	**0.020**
SUA after 2 days interval (mg/dL)	6.3 (0.8)	6.6 (1.0)	7.2 (1.4)	**0.019**
Increase in bodyweight over 2 days (kg)	1.94 (0.84)	1.96 (0.59)	2.25 ((0.79)	0.551
Changed hematocrit over 2 day (%)	2.74 (2.25)	2.72 (1.97)	3.19 (3.78)	0.861
Increase in SUA over 2 days (mg/dL)*^1^	4.35 (0.74)	4.44 (0.71)	4.83 (1.09)	0.239
Increase in SUA_Ht_ over 2 days (mg/dL)*^2^	4.56 (0.85)	4.65 (0.80)	5.12 (1.34)	0.223
**Hemodialysis with a 3-day interval**				
Pre-hemodialysis SUA (mg/dL)	6.0 (0.8)	6.4 (0.9)	6.9 (1.2)	**0.004**
Post-hemodialysis SUA (mg/dL)	1.6 (0.4)	1.7 (0.5)	1.9 (0.6)	0.139
SUA after 3 days interval (mg/dL)	7.1 (1.0)	7.7 (1.0)	8.4 (1.6)	** < 0.001**
Increase in bodyweight over 3 days (kg)	2.72 (1.06)	2.90 (0.97)	3.55 (1.67)	0.106
Changed hematocrit over 3 days (%)	3.29 (2.73)	3.57 (2.49)	4.73 (4.05)	0.361
Increase in SUA over 3 days (mg/dL)*^1^	5.46 (0.88)	5.99 (0.88)	6.51 (1.30)	** < 0.001**
Increase in SUA_Ht_ over 3 days (mg/dL)*^2^	5.77 (1.01)	6.35 (1.00)	7.10 (1.71)	** < 0.001**

Significant values are in bold.

The values represent the mean (standard deviation).

SUA*^1^: Unadjusted increase in serum urate levels.

SUA_Ht_*^2^: Increase in serum urate levels adjusted by hematocrit were calculated using the following formula: increase in SUA_Ht_ = unadjusted increase in SUA × [(100−Hematocrit)/(100−Hematocrit_post-HD_)].

### Extra-renal ABCG2 contribution to overall urate excretion and daily inflow into the uric acid pool

**Figure 2:**
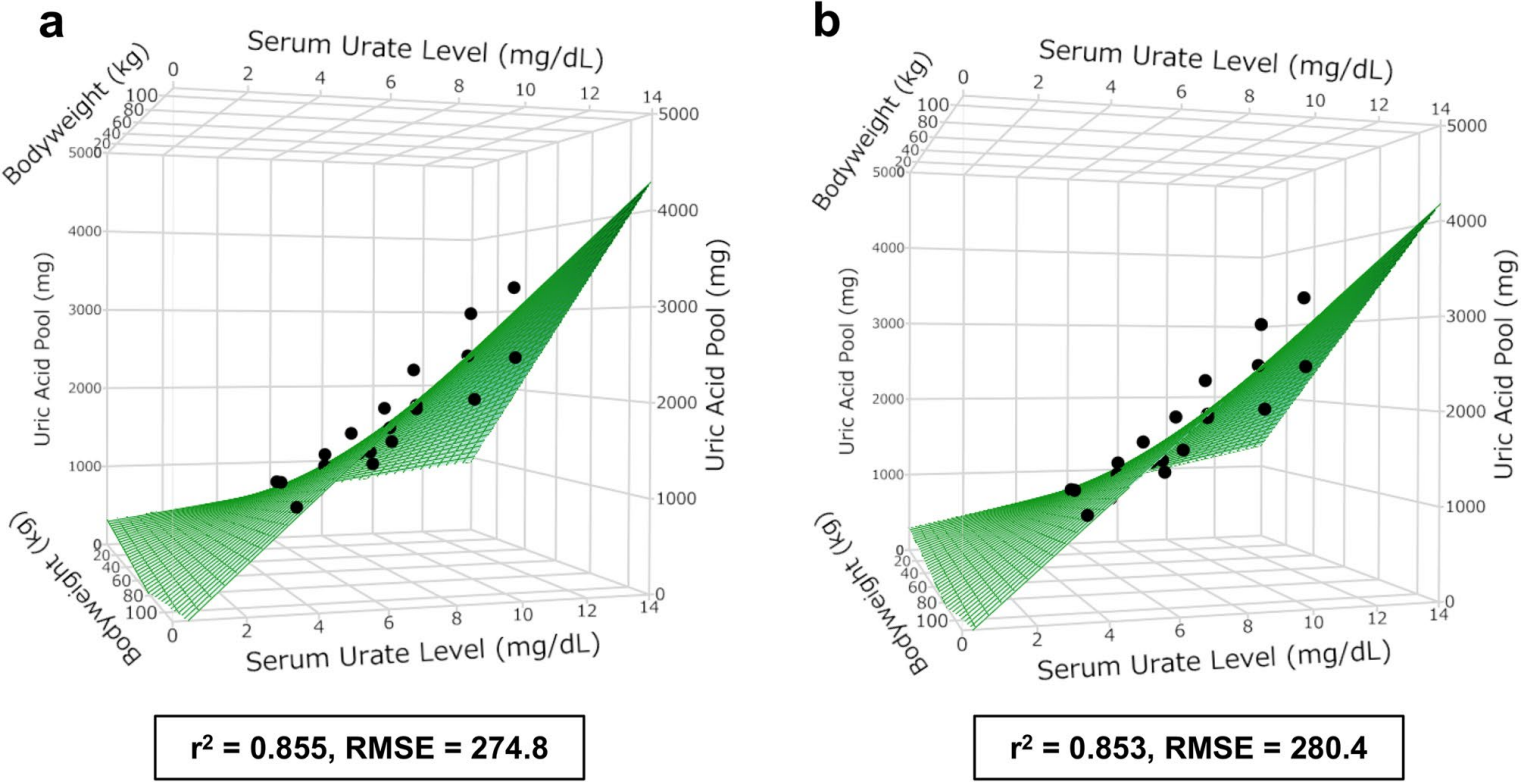
Conversion model of serum urate level adjusted by hematocrit (SUA_Ht_) and bodyweight to uric acid pool size derived from data for (**a**) adults and (**b**) adult males only in previous radio-isotopic studies. (**a**) Uric acid pool (mg) = 231.3 × SUA_Ht_ (mg∕dL) + 15.1 × bodyweight (kg) + [(SUA_Ht_−7.763) × (Bodyweight−75.55)] × 2.476−1131, r^2^ = 0.855, RMSE = 274.8. (b) Uric acid pool (mg) = 235.9 × SUA_Ht_ (mg∕dL) + 12.72 × bodyweight (kg) + [(SUA_Ht_−7.652) × (bodyweight−77.55)] × 2.116−961.8, r^2^ = 0.853, RMSE = 280.4.

To evaluate the contribution of extra-renal ABCG2 to the overall urate excretion and daily production of uric acid, we estimated Pool_UA_ from each participant’s SUA and bodyweight using conversion equations developed from the data in 3 previous radio-isotope studies using the least squares method considering interaction between SUA and bodyweight^7,11,12^.. Figure 2 and the following formula show the equation model derived from (a) mixed-sex adults (age > 15 years), and (b) adult males (age > 15 years) only:

$$\left({\varvec{a}}\right)\boldsymbol{ }{\varvec{M}}{\varvec{o}}{\varvec{d}}{\varvec{e}}{\varvec{l}}\boldsymbol{ }\,1:\boldsymbol{ }\boldsymbol{ }{\varvec{U}}{\varvec{r}}{\varvec{i}}{\varvec{c}}\boldsymbol{ }\,{\varvec{a}}{\varvec{c}}{\varvec{i}}{\varvec{d}}\boldsymbol{ }\,{\varvec{p}}{\varvec{o}}{\varvec{o}}{\varvec{l}}\boldsymbol{ }\,\left({\varvec{m}}{\varvec{g}}\right)=231.3\times {{\varvec{S}}{\varvec{U}}{\varvec{A}}}_{{\varvec{H}}{\varvec{t}}}\left({\varvec{m}}{\varvec{g}}/{\varvec{d}}{\varvec{L}}\right)+15.1\times {\varvec{B}}{\varvec{o}}{\varvec{d}}{\varvec{y}}{\varvec{w}}{\varvec{e}}{\varvec{i}}{\varvec{g}}{\varvec{h}}{\varvec{t}}\boldsymbol{ }\left({\varvec{k}}{\varvec{g}}\right)+\left({{\varvec{S}}{\varvec{U}}{\varvec{A}}}_{{\varvec{H}}{\varvec{t}}}-7.763\right)\times \left({\varvec{B}}{\varvec{o}}{\varvec{d}}{\varvec{y}}{\varvec{w}}{\varvec{e}}{\varvec{i}}{\varvec{g}}{\varvec{h}}{\varvec{t}}-75.55\right)\times 2.476-1131.$$

$$\left({\varvec{b}}\right)\boldsymbol{ }{\varvec{M}}{\varvec{o}}{\varvec{d}}{\varvec{e}}{\varvec{l}}\boldsymbol{ }\,2:\boldsymbol{ }\boldsymbol{ }{\varvec{U}}{\varvec{r}}{\varvec{i}}{\varvec{c}}\boldsymbol{ }\,{\varvec{a}}{\varvec{c}}{\varvec{i}}{\varvec{d}}\boldsymbol{ }\,{\varvec{p}}{\varvec{o}}{\varvec{o}}{\varvec{l}}\boldsymbol{ }\,\left({\varvec{m}}{\varvec{g}}\right)=235.9\times {{\varvec{S}}{\varvec{U}}{\varvec{A}}}_{{\varvec{H}}{\varvec{t}}}\left({\varvec{m}}{\varvec{g}}/{\varvec{d}}{\varvec{L}}\right)+12.72\times {\varvec{B}}{\varvec{o}}{\varvec{d}}{\varvec{y}}{\varvec{w}}{\varvec{e}}{\varvec{i}}{\varvec{g}}{\varvec{h}}{\varvec{t}}\boldsymbol{ }\left({\varvec{k}}{\varvec{g}}\right)+\left({{\varvec{S}}{\varvec{U}}{\varvec{A}}}_{{\varvec{H}}{\varvec{t}}}-7.652\right)\times \left({\varvec{B}}{\varvec{o}}{\varvec{d}}{\varvec{y}}{\varvec{w}}{\varvec{e}}{\varvec{i}}{\varvec{g}}{\varvec{h}}{\varvec{t}}-77.55\right)\times 2.116-961.8.$$

**Figure 3:**
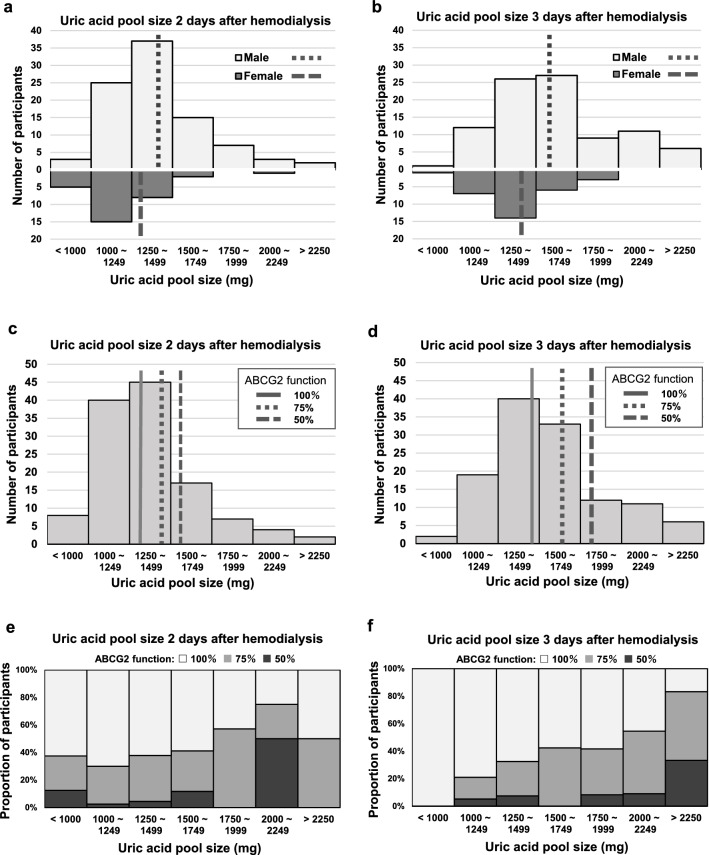
(**a**) White and gray histograms represent the uric acid pool after a 2-day interval in male and female participants, respectively. Dotted and a dashed lines represent the mean uric acid pool for males (mean ± standard deviation: 1410 ± 305 mg) and females (1223 ± 242 mg) after 2-day interval. (**b**) White and gray histograms represent the uric acid pool after a 3-day interval in male and female participants, respectively. Dotted and dashed lines represent the mean uric acid pool for males (1619 ± 365 mg) and females (1405 ± 245 mg) after a 3-day interval. (**c**) Uric acid pool size after a 2-day hemodialysis interval was 1363 ± 305 mg in overall participants. Solid, dotted, and dashed lines represent the mean of uric acid pool size in ABCG2 100, 75 and 50% functional groups, respectively [ABCG2 100% function (solid line), 1322 ± 283 mg; 75% function (dotted line), 1410 ± 314 mg; 50% function (dashed line), 1510 ± 418 mg (P = 0.129)]. (**d**) Uric acid pool size after a 3-day hemodialysis interval. The mean and standard deviation of the uric acid pool size in overall participants was 1565 ± 350 mg. Solid, dotted, and dashed lines represent the mean of the uric acid pool size in ABCG2 100, 75 and 50% functional groups, respectively [ABCG2 100% function (solid line), 1492 ± 313 mg; 75% function (dotted line), 1657 ± 337 mg; 50% function (dashed line), 1815 ± 545 mg (P = 0.006)]. (**e**) Relationship between ABCG2 dysfunction (75% and 50%) and uric acid pool size after a 2-day hemodialysis interval (P for trend = 0.071). (**f**) Relationship between ABCG2 dysfunction (75% and 50%) and uric acid pool size after a 3-day hemodialysis interval (P for trend = 0.002).

Unitless numbers (e.g., 231.3, 1131 and 7.763) in the conversion formulas in the manuscript are numbers that describe coefficients, intercept, and interactions. Model 1 was applied to mixed-sex (n = 123) and only female (n = 31) hemodialysis patient populations and Model 2 was applied to an only male (n = 92) hemodialysis patient population in this study. We applied the conversion equation to SUA_Ht_ after 2-day and 3-day hemodialysis intervals to estimate Pool_UA_. The means ± standard deviation of Pool_UA_ after 2-days and 3-day hemodialysis intervals were 1363 ± 305 mg (male, 1410 ± 310 mg; female, 1223 ± 242 mg) and 1565 ± 350 mg (male, 1619 ± 365 mg; female, 1405 ± 245 mg) (Fig. 3a,b).

Pool_UA_ after a 2-day interval was 1322 ± 283 mg, 1410 ± 314 mg, and 1510 ± 418 mg in 100, 75 and 50% ABCG2 functional groups (P = 0.129), respectively (Fig. 3c). Pool_UA_ after 3-day interval was 1492 ± 313 mg, 1657 ± 337 mg, and 1815 ± 545 mg in 100, 75 and 50% ABCG2 functional groups (P = 0.006), respectively (Fig. 3d). Figure 3e,f showed the proportions of participants with 100, 75 and 50% functional ABCG2 by the graded Pool_UA_ category (3e, after the 2-day interval; 3f, after the 3-day interval). Participants with dysfunctional ABCG2 (75 and 50%) tended to have larger Pool_UA_, and the trend was more robust after the 3-day interval (P for trend = 0.002) compared to the case of the 2-day interval (P for trend = 0.071).

**Figure 4:**
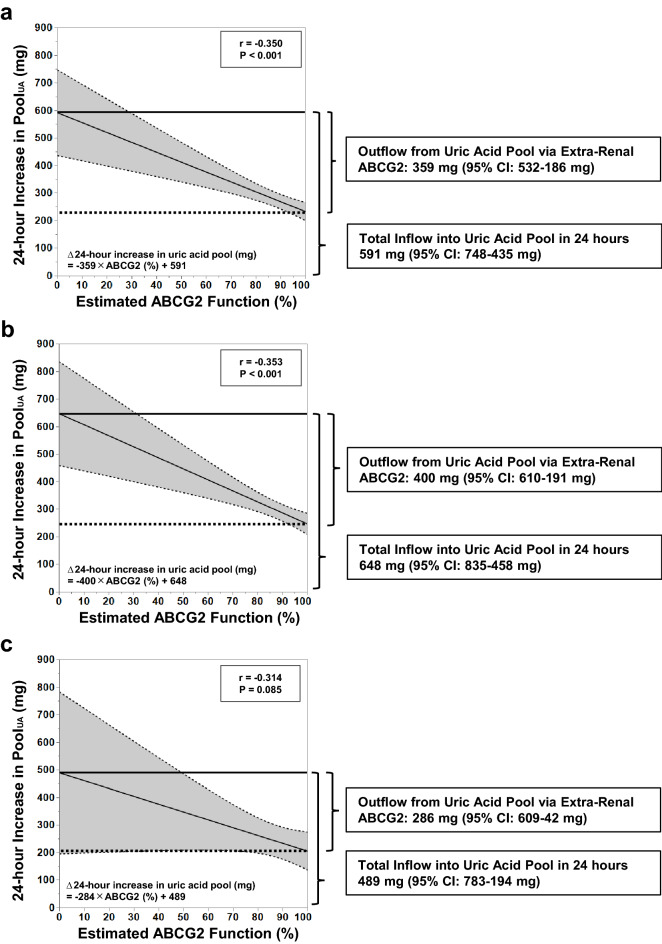
Contribution of extra-renal ABCG2 to urate excretion. The solid line shows the estimated regression for increase in uric acid pool per 24 h, and dotted lines show the 95% confidence interval of the regression line. (**a**) The regression model was derived from all hemodialysis participants (n = 123). The outflow/inflow of uric acid from/into the uric acid pool is 60.7%, and the ratio indicates the contribution of ABCG2 in urate excretion. (**b**) The regression model was derived from male hemodialysis participants (n = 92). The outflow/inflow ratio is 62.9%. (**c**) The regression model was derived from female hemodialysis participants (n = 31). The outflow/inflow ratio is 58.0%.

Figure 4 shows regression lines between ABCG2 function and the 24-h increase in Pool_UA_. The intercepts of the regression lines were 591 mg/day [95% confidence interval (95% CI) 748–435], 648 mg/day (95% CI 835–458), and 489 mg/day (95% CI 783–194) in all, male, and female participants, respectively. The intercepts represent the estimated 24-h increase in Pool_UA_ when ABCG2 was 0%. Therefore, they indicate an increase in Pool_UA_ approximately equal to that caused by the daily inflow into the Pool_UA_ because almost all extra-renal urate excretion is thought to exclusively depend on ABCG2. The slopes of the regression lines were 359 mg/day (95% CI 532–186), 400 mg/day (95% CI 610–191), and 286 mg/day (95% CI 609–42) in all, only male, and only female participants, respectively. The slopes represent the amount of efflux from Pool_UA_ by a fully functional ABCG2.

## Discussion

The kidney has been recognized as the main urate excretory organ, which is verified by the fact that SUAs are increased in renal failure due to a decreased renal uric acid excretory ability. On the other hand, urate excretion via the extra-renal pathway had been regarded as unrelated to SUA. Accordingly, hyperuricemia had been classified as ‘overproduction type’ or ‘underexcretion type’ based on only renal urate excretion^34^. In contrast, our previous study newly identified ‘extra-renal underexcretion type’ caused by ABCG2 dysfunction as the pathogenic concept underlying hyperuricemia^26^. This has advocated the importance of extra-renal urate excretion, which had been disregarded in urate dynamics.

In this study, SUAs of all participants were increased between the post-hemodialysis period and the subsequent hemodialysis session owing to the loss of kidney function, and ABCG2 dysfunction noticeably accelerated the increase in SUA (Table 2). The increasing SUA (i.e. increasing Pool_UA_) was synonymous with the urate excretion capacity being less than the uric acid production, and the acceleration of the increase in SUA implied that extra-renal ABCG2 contributed significantly to urate excretion. In addition, we attempted to assess Pool_UA_, but direct measurement of Pool_UA_ was not possible because of the inability to administer radio-isotopes. When radio-isotopes were not used, it was necessary to collect the secreted uric acid in the intestinal lumen to evaluate intestinal urate excretion and Pool_UA_. However, the collection of uric acid in the intestine is very difficult because uric acid is decomposed by uricase in the intestinal flora. Therefore, we firstly surveyed previous radio-isotope studies to investigate the relationship between SUA, bodyweight, and Pool_UA_^7,11,12^. Since no equation for conversion to Pool_UA_ had been reported so far, we then created the equations for the converting Pool_UA_ from individual SUA and bodyweight. Based on previous radio-isotope studies, a common reference has been that Pool_UA_ of a healthy male is approximately 1200 mg. However, the converting equation developed in our study enabled a tailor-made Pool_UA_ estimation. As a result, Pool_UA_ after 2-day and 3-day hemodialysis intervals was 1410 ± 310 mg and 1619 ± 365 mg in males, and 1223 ± 242 mg and 1405 ± 245 mg in females, respectively (Fig. 3a,b). These were comparable to or greater than the average Pool_UA_ of approximately 1200 mg (range: 937 to 1650 mg) and 600 mg (range: 541–687 mg) in healthy male and female individuals measured half century ago^1,3–5,7,8,11,12^, although the situations of hemodialysis patients differ from those of healthy individuals, such as the presence of artificial uric acid removal and abolition of renal function. Normally, it is well-known that there are sex differences in SUA in healthy individuals with residual renal function. SLC22A12, a uric acid reabsorption transporter, strongly regulates the urate excretion in the kidney and is upregulated by testosterone and downregulated by estrogen^35^, whereas it has been reported that intestinal ABCG2 expression does not differ markedly by sex^36^. Therefore, it is likely that there was no marked sex difference in SUA (male SUA: 7.4 ± 1.1 mg/dL, female SUA: 7.3 ± 1.1 mg/dL) in this study because anuric hemodialysis patients were subjected and they were deficient in the mechanism of uric acid reabsorption by SLC22A12 in the kidney. The slight sex differences in the Pool_UA_ can be attributed to the differences in the distribution volume of uric acid, such as differences in fat mass and muscle mass between males and females.

Additionally, ABCG2 dysfunction strongly contributed to an accumulation of uric acid and a larger Pool_UA_ (Fig. 3c–f), and the contribution due to ABCG2 dysfunction was more pronounced over a 3-day hemodialysis interval compared to a 2-day interval. Based on the association between the 24-h increases in Pool_UA_ and ABCG2 function, extra-renal urate excretion via ABCG2 was estimated to be equivalent to approximately 60% of the uric acid production (Fig. 4). Upregulation of Abcg2 expression in the ileum has been observed in 5/6 nephrectomized rats^28^. In the present study, it was inferred that in humans as in the rat model, loss of renal function up-regulated ABCG2 expression and increased extra-renal urate excretion. In other words, extra-renal ABCG2 can excrete up to approximately 60% of the uric acid production in response to decreased renal function. Therefore, ABCG2-mediated urate excretion in the intestine would serve an important role in compensating for the loss of renal urate excretion under conditions of decreased or lost renal function.

Uric acid is produced from hypoxanthine and xanthine by xanthine oxidase (also known as xanthine oxidoreductase) (XO). The XO activity in a human liver has been reported to be greater in male than in female, with large individual variations^37^. Regarding the study’s secondary aim, we estimated that 591 mg/day (male, 648 mg/day; female, 489 mg/day) of uric acid was produced and flowed into Pool_UA_ per 24 h. Our results may reflect sex differences in XO activity, which have not been validated within this study. Although the hemodialysis patients had dietary restrictions such as limits on water, sodium, phosphorus, and potassium intake, their purine intake was not restricted. Additionally, there were no limitations on protein intake, which fluctuates consistently with purine intake. In light of these details, our results are applicable to the general population with regular daily dietary habits, except for excessive drinking and eating. The result represents the first update on the daily amount of uric acid production in more than 50 years.

However, several limitations must be acknowledged. (1) Since this study design is non-interventional, our analyses were based on SUA at only three time points: before and at the end of hemodialysis and 2 days or 3 days after the hemodialysis. Therefore, detailed SUA variations remained unidentified. In addition, this study did not directly measure the amount excreted from the intestinal tract, and this needs to be verified as a future issue. (2) None of the participants had severely dysfunctional ABCG2 (i.e., 25 or 0%). Further studies should recruit participants with severe ABCG2 dysfunction. (3) We only estimated ABCG2 function by genotyping using 2 common SNPs (p.Q126X and p.Q141K) and did not directly analyze the actual ABCG2 transport activity. Therefore, we need to understand that the influence of rare variants remains although these 2 common variants can explain most of the ABCG2 function and that the genotypes are not essentially the same as the real ABCG2 efflux capacity in the given subjects. Recently, a PET-based method has been reported as a direct method to measure the transport activity of ABCG2^38^. A less invasive method is to measure the expression level of ABCG2 from collected red blood cells^39^. The advantage of our method for estimating the ABCG2 function is less invasive and more clinically applicable because it uses genomic DNA collected from blood and saliva. Direct measurement of ABCG2 transport activity is the future issue to validate the results of this study. (4) There was no independent dataset for validation and the population in the present study was relatively small. In order to assess the robustness of our results, it is necessary to conduct validation studies in a population large enough to capture a sufficient number of such individuals. (5) Because of the assumption that ABCG2 is a predominant transporter in the extra-renal urate excretion, the effects of other transporters such as SLC17A4 were determined to be few and were not considered in the estimation of daily uric acid production in this study. Consequently, it is possible that the estimated daily uric acid production was slightly underestimated.

In conclusion, it was estimated that approximately 650 mg or 500 mg of uric acid was produced daily in male or female hemodialysis participants, 60% of which was excreted via ABCG2 in the extra-renal pathway. Our findings highlight the critical importance of extra-renal excretion in the homeostasis of SUA.

## Materials and methods

### Study design and participants

The present study was designed as a non-interventional observation study with each participant’s estimated ABCG2 function as a predictor and change in SUA as an outcome. The study was conducted using clinical data before and after hemodialysis. To estimate the amount of extra-renal excretion and daily production of uric acid from the outcome, we defined our study population as anuric hemodialysis patients. The 236 original candidates were recruited at 3 hemodialysis outpatient units from 2018 to 2019. They were scheduled for regular hemodialysis (4 h/session) 3 times a week. This study used the following exclusion criteria. Patients with missing values (n = 8) and ages older than 80 years (n = 23) were excluded. Patients with high parathyroid hormone (> 300 pg/mL, n = 24) were excluded because parathyroid hormone down-regulates ABCG2 expression in secondary hyperparathyroidism^40^. ABCG2 is associated with hyperuricemia and gout^30,41–43^, and uric acid-lowering medication would suppress ABCG2 function^44^. Additionally, a secondary objective of this study was to estimate the daily amount of uric acid production. Therefore, patients using xanthine oxidoreductase (xanthine oxidase) inhibitors (n = 58) were excluded. Ultimately, 123 hemodialysis participants were included in this study. The flowchart for the present study is summarized in Fig. 1.

### Data collection

**Figure 5:**
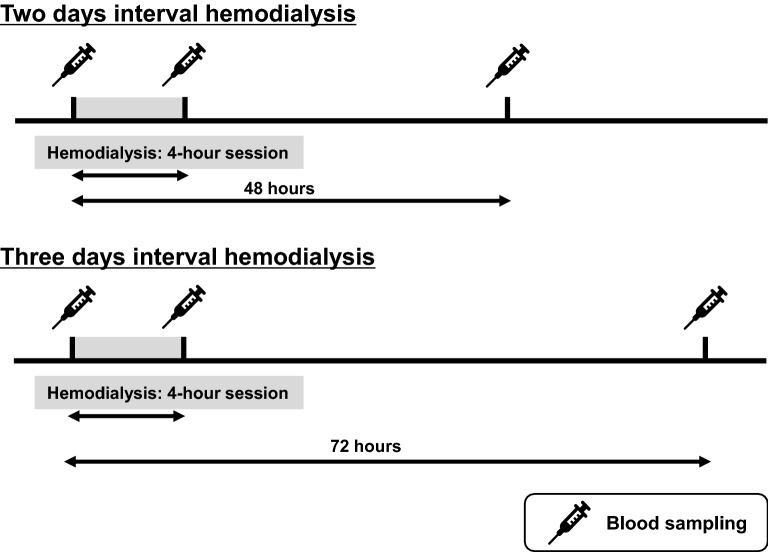
Study design for blood sampling.

Age, sex, height, bodyweight, body mass index, and systolic and diastolic blood pressure were obtained from medical records at the study entry. We performed blood sampling pre-hemodialysis, post-hemodialysis, and before the subsequent hemodialysis after a 2-day interval (44 h from post-hemodialysis) or 3-day interval (68 h from post-hemodialysis) (Fig. 5). The total of the three blood data per hemodialysis session were collected from same participants and were evaluated as a within-subject parameter. Red blood cell counts, hematocrit, hemoglobin, platelet, serum albumin, blood urea nitrogen, serum creatinine, SUA, aspartate aminotransferase, alanine aminotransferase, gamma-glutamyl transpeptidase, parathyroid hormone, sodium, potassium, chloride, and phosphate levels were measured.

### SNP genotyping and ABCG2 functional categorization

The whole procedures were performed anonymously. About 5 mL of the blood samples collected from each participant were centrifuged to isolate leukocytes. Thereafter, a lysis buffer containing 3 μg/mL proteinase K was added to the cells. After extraction with TE saturated phenol, ethanol precipitation was performed to purify the genomic DNA. We carried out TaqMan-based probe qualitative real-time PCR assay for genotyping using a THUNDERBIRD Probe qPCR Mix kit (Toyobo, Osaka, Japan) and PikoReal 96 system (Thermo Fisher Scientific, Waltham, Massachusetts), according to the manufacturer’s protocol with the extracted genomic DNA. PCR was performed using the probes fluorescently labeled with HEX and FAM. We used primers for rs72552713 (forward and reverse primer sequences were 5’-GCAAGGAAAGATCCAAGTGG-3’ and 5’-ATCAGCCAAAGCACTTACCC-3’, respectively) and rs2231142. (forward and reverse primer sequences were 5’-AGGATGATGTTGTGATGGGC-3’ and 5’-ATTACCTTGGAGTCTGCCAC-3’, respectively). We evaluated the ABCG2 function of all participants by a genotyping a combination of 2 common SNPs, rs72552713 (c.376C > T, p.Q126X, risk allele: T) and rs2231142 (c.421C > A, p.Q141K, risk allele: A) (Supplementary Fig. S1). p.Q126X eliminates ABCG2 function completely, while p.Q141K halves ABCG2 function. p.Q126X and p.Q141K are independent risk factors for ABCG2 dysfunction because these SNPs are in different haplotypes^41^. Although several non-synonymous variants of *ABCG2* without p.Q126X and p.Q141K have been reported, p.Q126X and p.Q141K can explain most of the ABCG2 function because the other variants is extremely rare (risk allele frequency < 1%) in Japanese population^45^. The estimation of ABCG2 function is useful for various ABCG2-related analyses and it is able to categorize ABCG2 function into the following functional categories: 100, 75, 50, 25 and 0%^26,30,31,41–43,46^. In this study, the estimated ABCG2 function was divided into 3 categories: 100, 75 and 50%.

### Conversion of uric acid pool size from serum urate level and bodyweight

We calculated the increase in SUAs per 24 h based on the difference between SUA increases over 2-day and 3-day hemodialysis intervals. In anuric hemodialysis patients, fluid volumes were postulated to increase proportionally with water intake. In other words, drinking water dilutes serum urate and may lead to underestimation of elevated SUA as an effect modifier. Therefore, we adjusted the change in SUA and the fluid storage using the hematocrit proportion as shown in the following formula:

$${\varvec{A}}{\varvec{d}}{\varvec{j}}{\varvec{u}}{\varvec{s}}{\varvec{t}}{\varvec{e}}{\varvec{d}}\boldsymbol{ }\,{\varvec{c}}{\varvec{h}}{\varvec{a}}{\varvec{n}}{\varvec{g}}{\varvec{e}}\boldsymbol{ }\,{\varvec{i}}{\varvec{n}}\boldsymbol{ }\,{\varvec{S}}{\varvec{U}}{\varvec{A}}\boldsymbol{ }\,\left({{\varvec{c}}{\varvec{h}}{\varvec{a}}{\varvec{n}}{\varvec{g}}{\varvec{e}}\boldsymbol{ }\,{\varvec{i}}{\varvec{n}}\boldsymbol{ }\,{\varvec{S}}{\varvec{U}}{\varvec{A}}}_{{\varvec{H}}{\varvec{t}}}\boldsymbol{ }\,[{\varvec{m}}{\varvec{g}}/{\varvec{d}}{\varvec{L}}]\right)=({\varvec{S}}{\varvec{U}}{\varvec{A}}-{{\varvec{S}}{\varvec{U}}{\varvec{A}}}_{{\varvec{p}}{\varvec{o}}{\varvec{s}}{\varvec{t}}\boldsymbol{ }{\varvec{H}}{\varvec{D}}})\times (100-{\varvec{H}}{\varvec{t}}[\boldsymbol{\%}])/(100-{{\varvec{H}}{\varvec{t}}}_{{\varvec{p}}{\varvec{o}}{\varvec{s}}{\varvec{t}}\boldsymbol{ }{\varvec{H}}{\varvec{D}}}[\boldsymbol{\%}])$$

Pool_UA_ was estimated from SUA and bodyweight using an equation that we developed based on SUA, bodyweight, and Pool_UA_ in previous radio-isotope experimental papers using least squares method considering interaction between SUA and bodyweight^7,11,12^.

### Statistical analysis

Continuous variables are presented as means and standard deviation, and binary variables are presented as percentages in the summary of patient characteristics for each ABCG2 category. Differences among continuous and binary variables were determined by analysis of variance (ANOVA) or Fisher’s exact test at the 5% significance level. In addition, the relationship between ABCG2 dysfunction (75 and 50% functional ABCG2) and Pool_UA_ was analyzed by two-tailed Cochran-Armitage trend test. Although uric acid is excreted via both renal and extra-renal pathways, urate excretion in anuric hemodialysis patients virtually depended on ABCG2 in the extra-renal pathway because their renal pathway was disrupted. Therefore, we calculated the daily amount of uric acid production and daily extra-renal urate excretion by a regression line between the 24-h increase in uric acid pool and estimated ABCG2 function. The linear regression analysis was performed based on the least squares method and the intercept, slope, and 95% confidence intervals were calculated. For all calculations in the statistical analysis, software JMP Pro 15 (SAS Institute Inc., Cary, North Carolina) was used.

### Ethical considerations

The present study was conducted in accordance with the Declaration of Helsinki. The epidemiological survey was approved by the Institutional Review Board (IRB)/Ethics Committee of Tokyo University of Pharmacy and Life Sciences (Approval #16-32) and the Tokai University (Approval #16-52). Written informed consent was obtained from all participants at the beginning of the study, and participants were allowed to discontinue.

## Supplementary Information

Supplementary Figure 1.

## Data Availability

The original data that support the findings of this study are available on request to the corresponding author. The data are not publicly available due to restrictions e.g. their containing information that could compromise the privacy of research participants.
