# Supplementary material for: Evaluation of ABCG2-mediated extra-renal urate excretion in hemodialysis patients
Source: Sci Rep. 2023 Jan 13;13:93. doi: 10.1038/s41598-022-26519-x (PMC9839766; doi:10.1038/s41598-022-26519-x)
Supplement: Supplementary file 1 — Supplementary Figure 1. [file 41598_2022_26519_MOESM1_ESM.pdf]

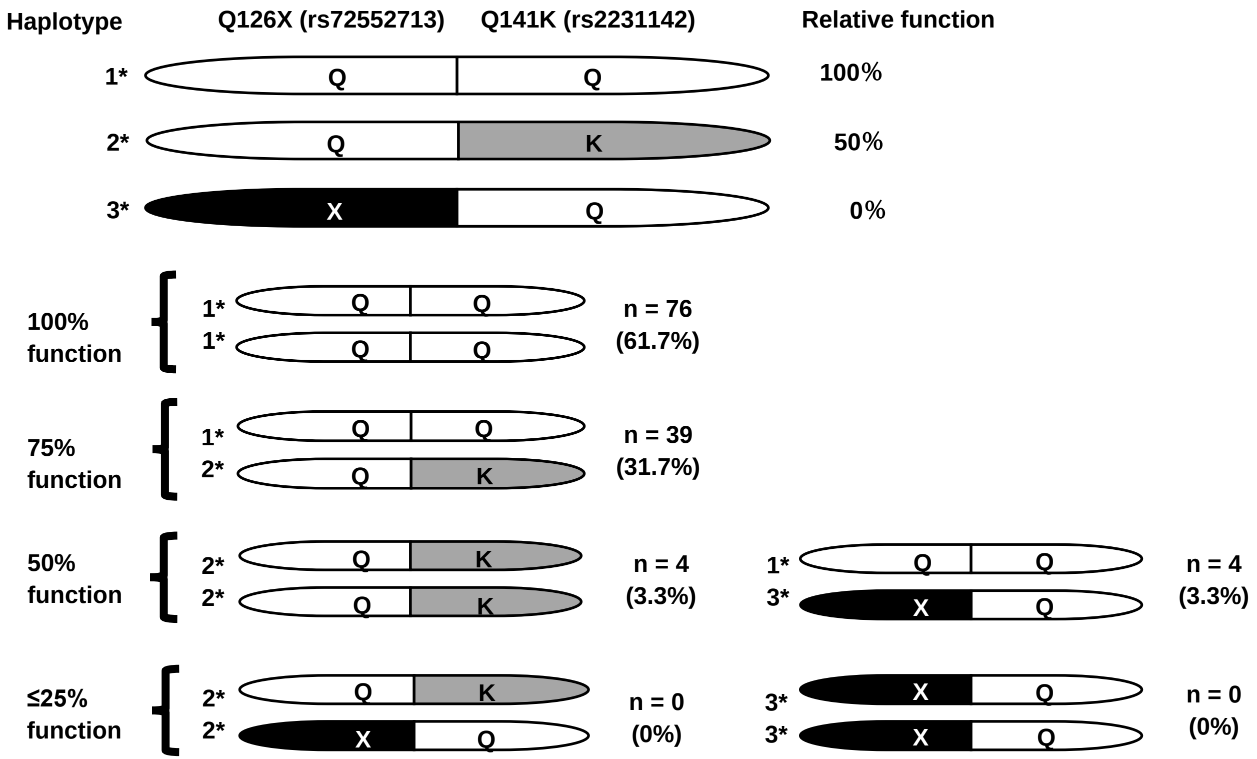

Supplementary Figure 1. Estimation of ABCG2 function using genotype combination of two common single nucleotide polymorphisms. The ABCG2 function was evaluated based on combining rs72552713 (c.376C > T, p.Q126X, risk allele: T) and rs2231142 (c.421C > A, p.Q141K, risk allele: A). All participants were classified into ABCG2 100% (61.7%), 75% (31.7%), 50% (6.6%) functional groups by the genotype combination.
